# Charge Compensation-Directed Enhanced Photoluminescence in M^+^ (M = Li, Na, K) Co-Doped Novel Red Phosphor Ca_2.5_Hf_2.5_Ga_3_O_12_:Eu^3+^ for Lighting Applications

**DOI:** 10.3390/molecules31132397

**Published:** 2026-07-07

**Authors:** Hua Li, Zijun Huang, Yifei Hou, Qiyue Liu, Di Li, Wenyue Zhang, Yi Su, Zhide Wang, Zaifa Yang

**Affiliations:** College of Physics and Electronic Engineering, Qilu Normal University, Jinan 250200, China; hzj18765157511@163.com (Z.H.); houyifei060808@163.com (Y.H.); liu19063412601@163.com (Q.L.); sevensto1115@163.com (D.L.); zhang111120231122@163.com (W.Z.); suyi051115@163.com (Y.S.); zhidewang44@gmail.com (Z.W.)

**Keywords:** phosphor, charge compensators, WLED

## Abstract

Against the backdrop of energy conservation and environmental protection, developing more stable and efficient phosphors has become an urgent challenge. In this study, we have synthesized a series of Ca_2.5_Hf_2.5_Ga_3_O_12_:Eu^3+^ (CHGO:Eu^3+^) red phosphors via a high-temperature solid-state method, which exhibit strong red emission at 610 nm under 394 nm excitation, corresponding to the ^5^D_0_→^7^F_2_ transition of Eu^3+^. To improve the lattice vacancies caused by charge imbalance when Eu^3+^ is doped into the CHGO lattice to replace Ca^2+^, we introduce the charge compensator M^+^ (M = Li, Na, K). The results of the emission spectrum show that the introduction of charge compensators can effectively improve the luminescence intensity. Among them, K^+^ has the most significant effect on increasing the emission intensity of Eu^3+^, making the emission intensity of the phosphor more than twice that when there are no charge compensation ions. Additionally, the quantum efficiency and thermal stability of these phosphors are significantly improved compared to the CHGO:0.075Eu^3+^ sample before substitution. At 423 K, the emission intensity of the CHGO:0.075Eu^3+^, 0.075K^+^ sample still remains at 88.7% of that at 298 K. The color rendering index of the prepared white LED is 81.2, and its CIE chromaticity coordinates are (0.3212, 0.3065). This indicates that the prepared CHGO:0.075Eu^3+^, 0.075K^+^ red phosphor has broad application prospects in solid-state lighting.

## 1. Introduction

White light-emitting diodes (WLEDs) have become some of the most valuable new light sources because of their remarkable energy-saving characteristics and satisfactory economy [1,2]. Traditional WLEDs are usually composed of blue chips and yellow-emitting Y_3_Al_5_O_12_:Ce^3+^ (YAG:Ce^3+^) phosphor [3]. However, due to the lack of a red component, WLEDs show a low color rendering index (CRI) and high color temperature (CCT) [4,5]. WLEDs, which are made by combining green, red and blue phosphors with near-ultraviolet (NUV) LED chips, can solve these problems well. Under this background, it has become the focus of research to find new red phosphors that can be excited by NUV light and can effectively improve their luminescence intensity.

As a typical activator of red luminescent materials, the absorption of Eu^3+^ at about 394 nm can be well matched with that of NUV chips [6]. Compared with nitrides and fluorides, Eu^3+^-activated oxide red phosphors not only have the advantages of environmental friendliness, but also have the characteristics of simple preparation, high thermal stability and strong corrosion resistance [7,8]. In addition, the matrix materials also have an important influence on the photoluminescence properties of the phosphors. The garnet structure has attracted much attention because of its excellent thermal stability and low phonon energy, which is helpful to avoid non-competitive non-radiative relaxation of rare-earth ions [9,10]. Therefore, it is regarded as one of the important substrates for photoluminescence applications. Among them, a new type of garnet oxide Ca_2.5_Hf_2.5_Ga_3_O_12_ (CHGO) has attracted wide attention from researchers because of its stable crystal structure, non-toxic, pollution-free, and simple preparation [11]. Chang et al. reported that the maximum absolute sensitivity of Dy^3+^-doped CHGO is 0.13% K-1, which indicates that CHGO:Dy^3+^ has excellent optical temperature measurement behavior in optical temperature measurement applications [12]. Yan et al. reported that broadband near-infrared emission centered at 770 nm has been realized in Fe^3+^-doped CHGO, which shows great potential in nondestructive testing applications [13]. Alkali metal ion M^+^ (M = Li, Na, K) has a low oxidation state and a special ion radius, which can improve the luminescence intensity and fluorescence efficiency of phosphors by charge compensation [14]. It has been widely used to improve the luminescence intensity of rare-earth-doped phosphors [15]. It is found that it can improve the crystallinity and particle size of phosphors and is beneficial to improve the photoluminescence intensity of phosphors [16].

Inspired by previous research results, a series of CHGO:xEu^3+^ phosphors, which can be effectively excited by NUV light, were designed and synthesized on the basis of CHGO. Considering the obvious difference in ion radius and charge between Eu^3+^ and Ca^2+^, the strategy of co-doping alkali metal ions M^+^ (M = Li, Na, K) was adopted. Compared with the samples doped with Na^+^/Li^+^ and undoped samples, it was observed that the fluorescence intensity was significantly enhanced after doping with K^+^. In addition, the intrinsic mechanism of the significant improvement in sample emission intensity, quantum efficiency, and thermal stability was systematically analyzed through lattice defect theory. At the same time, the application of the synthesized samples in the WLED field was also discussed.

## 2. Results and Discussion

### 2.1. Crystal Structure

**Figure 1:**
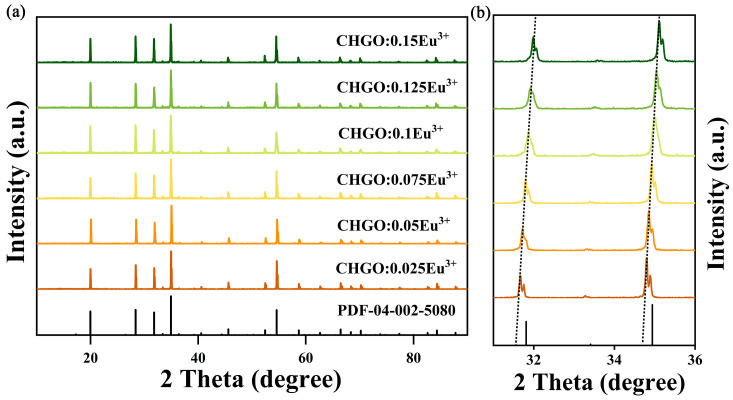
(**a**) XRD patterns of CHGO:xEu^3+^ (x = 0.025, 0.05, 0.075, 0.1, 0.125, 0.15) and (**b**) enlarged patterns corresponding to 31°–36°.

Figure 1a shows the XRD patterns of the CHGO:xEu^3+^ (x = 0.025, 0.05, 0.075, 0.1, 0.125, 0.15) samples, with the XRD standard card of CHGO (PDF # 04-002-5080) at the bottom. It can be seen from the figure that the diffraction peaks of CHGO:xEu^3+^ are consistent with those of the CHGO standard card, which indicates that the sample has high purity and excellent crystallization behavior, and also shows that the doping of Eu^3+^ will not destroy the crystal structure of CHGO. Figure 1b shows an enlarged view of the main diffraction peaks from 31° to 36°. With the increase in Eu^3+^ doping concentration, all diffraction peaks gradually move to a larger angle. This indicates that the interplanar spacing of phosphors decreases with the increase in Eu^3+^ ions, that is, Eu^3+^ ions may replace some ions with larger radii, resulting in lattice shrinkage [17]. According to the principle of ionic radius similarity, the possibility of ionic substitution can be judged by the radius percentage formula, and the relevant formula is as follows [18]:

(1) $$D_{r}=\frac{R_{m}(CN)-R_{d}(CN)}{R_{m}(CN)}$$

where D_r_ is the percentage of ionic radius difference, and R_m_ and R_d_ are the ionic radii of the substituted ions and substituted ions, respectively. The calculated results of D_r_ < 30% indicate that the ion radius is within a reasonable range, and the smaller the D_r_ value, the easier the substitution occurs [19]. The Dr values of Eu^3+^ substituted Ca^2+^ (CN = 8), Hf^4+^ (CN = 8) and Hf^4+^ (CN = 6) were 5.06%, 28.4% and 33.4%, respectively, which indicated that Eu^3+^ was easier to enter the Ca^2+^ lattice. Due to the unequal valence substitution of Ca^2+^ by Eu^3+^ incorporated into the CHGO lattice, according to the principle of defect formation, Ba^2+^ vacancies or interstitial oxygen will be generated to balance the charge. When the concentration of such defects is too high, it leads to lattice distortion and thus reduces the luminescence intensity. This also provides a pathway for us to explore the introduction of charge compensators to enhance the luminescence properties in the next step.

**Figure 2:**
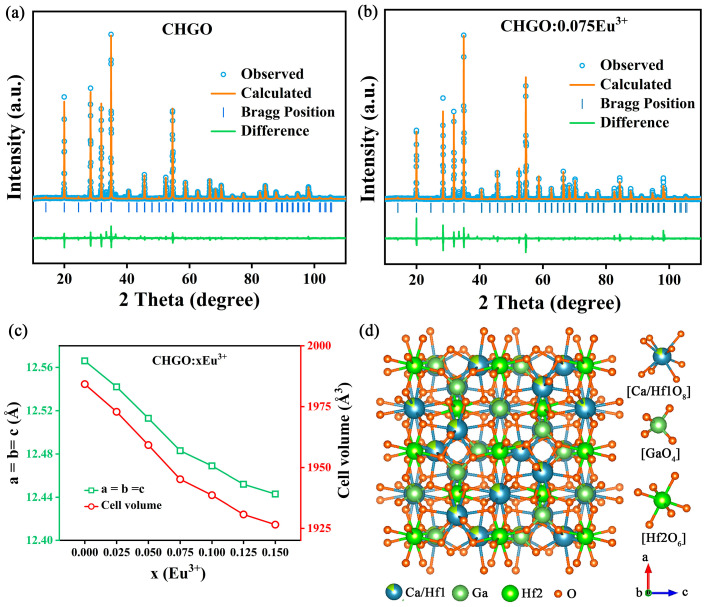
Rietveld refinement of (**a**) CHGO and (**b**) CHGO:0.075Eu^3+^. (**c**) Volume change in CaO_8_ and size change in parameters a/b/c. (**d**) The crystal structure of CHGO.

In order to further analyze the crystal structure of CHGO:xEu^3+^ (x = 0, 0.025, 0.05, 0.075, 0.1, 0.125, 0.15) samples, the XRD data of the best samples were refined by the General Structure Analysis System (GSAS) based on the crystallographic data of CHGO as the initial structure model [20]. Figure 2a,b show experimental data, calculated data, and corresponding XRD refinement differences for CHGO and CHGO:xEu^3+^ (x = 0.025, 0.05, 0.075, 0.1, 0.125, 0.15). According to the results of Table 1, the structure of the CHGO matrix and CHGO:0.075Eu^3+^ belongs to the cubic garnet structure. In addition, the confidence factors of CHGO (R_p_ = 5.71%, R_wp_ = 7.54%) and CHGO:0.075Eu^3+^ (R_p_ = 5.44%, R_wp_ = 8.02%) are relatively small, which indicates that the true crystallographic structure of the samples matches the crystallographic structure of the standard model [21]. Figure 2c shows that the lattice parameters (a = b = c) and unit cell volume (V) decrease with the introduction of Eu^3+^, as expected, thus further confirming the occurrence of lattice shrinkage. Figure 2d shows the crystal structure of CHGO. CHGO crystal has a garnet structure, and its space group is Ia-3d, which belongs to the cubic structure. In the CHGO structure, Ca, Hf and Ga coordinate with 8, 8/6 and 4 oxygen atoms, respectively, forming three coordination polyhedra, which are the [Ca/Hf1O_8_] dodecahedron, [GaO_4_] tetrahedron and [Hf2O_6_] octahedron. The [Ca/Hf1O_8_] dodecahedron, [GaO_4_] tetrahedron and [Hf2O_6_] octahedron are connected by edge sharing, and the [Hf2O_6_] octahedron and [GaO_4_] tetrahedron share a vertex angle, and then form a three-dimensional frame together. Because the radii of Ca^2+^ and Eu^3+^ ions are similar, we think that Eu^3+^ mainly replaces Ca^2+^ in the [Ca/Hf1O_8_] dodecahedron.

**Figure 3:**
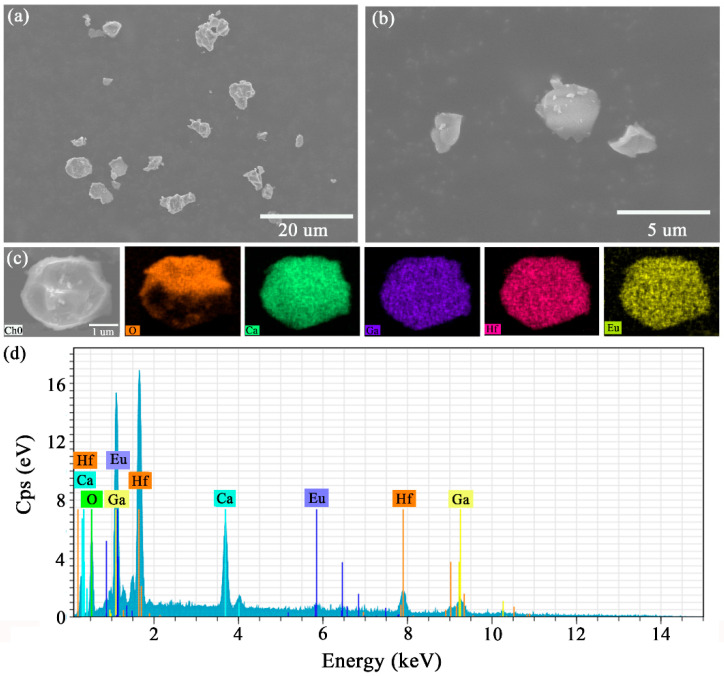
SEM images of CHGO:0.075Eu^3+^ phosphor with different magnifications: (**a**) 20 μm, (**b**) 5 μm. (**c**) The corresponding element mapping of Ca, Ga, Hf, O and Eu in CHGO:0.075Eu^3+^ phosphor. (**d**) The energy dispersion spectra of the CHGO:0.075Eu^3+^ phosphor.

In order to accurately characterize the microscopic distribution characteristics of the elements in the samples, SEM combined with EDS was used to characterize the element distribution of the samples. Figure 3a,b are microstructure diagrams of CHGO:0.075Eu^3+^ at different magnifications. It can be seen from the figures that the particle size of the sample is about 2~4 μm. They are uniform in size, well-defined in shape, and well-crystallized, with smooth surfaces. Agglomeration is also observed, which may be due to insufficient grinding or agglomeration occurring during the high-temperature sintering process [22]. Figure 3c is an SEM image of the element distribution of CHGO:0.075Eu^3+^. It can be seen that Ca, Hf, Ga, Eu and O, all five elements are detected and all elements are uniformly distributed, indicating that Eu^3+^ has been uniformly doped in the matrix lattice. The results of EDS energy spectrum analysis in Figure 3d show that only Ca, Hf, Ga, Eu and O elements are detected in the samples, and no other impurity elements are found. The results show that the CHGO:0.075Eu^3+^ sample has high chemical purity, providing experimental evidence for the successful doping of Eu^3+^ ions into the matrix lattice.

### 2.2. Optical Properties of CHGO:Eu^3+^

**Figure 4:**
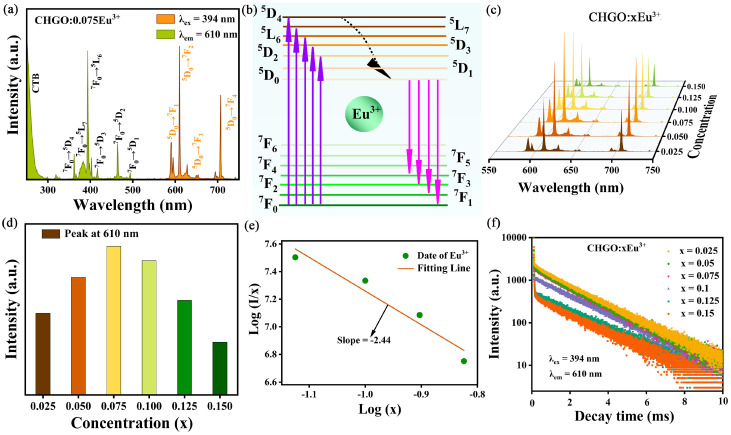
(**a**) The excitation and emission spectra of CHGO:0.075Eu^3+^ phosphor; (**b**) The energy-level diagram of Eu^3+^. (**c**) The emission spectra of CHGO:xEu^3+^ (x = 0.025, 0.05, 0.075, 0.1, 0.125, 0.15) phosphors. (**d**) The relationship between Eu^3+^ mole fraction and emission peak intensity at 610 nm. (**e**) Relationship of log(x) versus log(I/x) for CHGO:xEu^3+^. (**f**) Decay curves of CHGO:xEu^3+^.

Figure 4a shows the excitation spectrum of CHGO:0.075Eu^3+^ phosphor at the monitoring wavelength of 610 nm and the emission spectrum at the excitation wavelength of 394 nm. The strong and wide excitation band in the range of 250~300 nm is due to the charge transfer band (CTB) between Eu^3+^ and O^2−^, and this part of charge transfer absorption comes from the energy absorbed by electrons from the 2p orbital of O to the 4f empty orbital of Eu [23,24]. However, a series of peaks between 300 and 550 nm coincide with the characteristic excitation peaks of Eu^3+^, and the strong excitation peaks at 394 nm and 465 nm correspond to the ^7^F_0_→^5^L_6_ and ^7^F_0_→^5^D_2_ transitions of Eu^3+^, respectively, which also shows that the CHGO:0.075Eu^3+^ can be effectively excited by NUV light and blue light [25,26]. It can also be seen from Figure 4a that under the excitation of the strongest peak at 394 nm, the emission spectrum of the sample mainly consists of five emission peaks at 591, 610, 654 and 708 nm, respectively, which belong to the electronic transitions of ^5^D_0_→^7^F_1_, ^5^D_0_→^7^F_2_, ^5^D_0_→^7^F_3_ and ^5^D_0_→^7^F_4_ of Eu^3+^ [27]. Figure 4b is the energy level transition diagram of CHGO:0.075Eu^3+^, which shows the more detailed energy level transition process in excitation and emission. Figure 4c shows the emission spectra of CHGO:xEu^3+^ (x = 0.025, 0.05, 0.075, 0.1, 0.125, 0.15) phosphors with different Eu^3+^ doping concentrations. With the increase in Eu^3+^ doping concentration, the peak shape and position of the emission spectra do not change; only the intensity changes. Figure 4d shows the relationship between Eu^3+^ doping concentration and luminescence intensity. It can be seen from the figure that the emission intensity increases with the increase in Eu^3+^ concentration. When the doping concentration of Eu^3+^ is 0.075, the emission intensity is the highest, and when the concentration of Eu^3+^ is greater than 0.075, the emission intensity decreases, resulting in concentration quenching [28,29]. The non-radiative energy transfer mechanism leading to concentration quenching is mainly exchange and electric multipole interaction. Its action mechanism can be preliminarily judged by the critical distance (Rc), and the calculation formula is as follows [30]:

(2) $$R_{c}=2{\left(\frac{3V}{4\pi x_{c}N}\right)}^{1/3}$$

where x_c_, N and V are the critical concentration, cation number and unit cell volume of Eu^3+^, respectively. For the CHGO:0.075Eu^3+^ sample, x_c_ = 0.075, N = 8, V = 1945.17 Å^3^. The R_c_ value is 9.18 Å, which is greater than 5 Å, indicating that the concentration quenching mechanism of Eu^3+^ may be affected by the electric multipole interaction [31]. According to Dexter’s energy transfer theory of electrical multipole interaction, the type of interaction mechanism between Eu^3+^ can be evaluated by the following formula [32]:

(3) $$\frac{I}{x}=K{\left(1+\beta {\left(x\right)}^{Q/3}\right)}^{-1}$$

where Q values of 6, 8 and 10 correspond to the energy transfer mechanism of dipole–dipole, dipole–quadrupole and quadrupole–quadrupole interactions, respectively. Linear regression analysis is performed on lg (I/x)~lg (x), as shown in Figure 4e. The slope of the fitting line is −2.44, and the Q value is 7.32, which is closest to 8. It is confirmed that the dipole-quadrupole interaction is the main mechanism leading to concentration quenching in CHGO:xEu^3+^ samples.

Figure 4f shows the decay curves of the CHGO:xEu^3+^ series phosphors measured at 395 nm excitation wavelength, and fits them with a single exponential function:

(4) $$I_{t}=I_{0}+A\exp(\frac{-t}{\tau })$$

where I_t_ is the luminous intensity at time t. The decay lifetimes of CHGO:xEu^3+^ (x = 0.025, 0.05, 0.075, 0.1, 0.125, 0.15) were 1.23, 1.15, 0.98, 0.82, 0.65 and 0.60 ms, respectively, with the increase in Eu^3+^ concentration. This downward trend also provides favorable evidence for the increase in non-radiative energy transfer.

### 2.3. Effect of Charge Compensator M^+^ (M = Li, Na, K) on Luminescence Properties

When Eu^3+^ is doped into the CHGO lattice, Ca^2+^ is substituted by different valence states, and vacancies will be produced in the lattice, which will reduce the luminescence performance. In order to improve this phenomenon, the charge compensator M^+^ (M = Li, Na, K) can be introduced to compensate for the vacancy caused by Eu^3+^ doping. When M^+^ (M = Li, Na, K) is doped into the phosphor and charge compensation is carried out for Eu^3+^ substitution of Ca^2+^, according to the principle of defect formation, interstitial M^+^ (M = Li, Na, K) or oxygen vacancies will be generated to achieve electrical neutrality, as shown in the following equation:

(5) $$Eu_{2}O_{3}+MO_{2}+4CaO\to 2{{Bi}_{Ca}}^{•}+2{M_{Ca}}^{\prime }+4O_{0}$$

**Figure 5:**
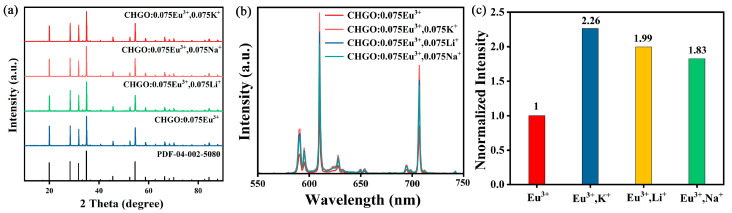
(**a**) XRD patterns of CHGO:0.075Eu^3+^ and CHGO:0.075Eu^3+^, 0.075M^+^ (M = Li, Na, K). (**b**) The emission spectra of CHGO:0.075Eu^3+^ and CHGO:0.075Eu^3+^, 0.075M^+^ (M = Li, Na, K). (**c**) The integrated emission intensity at 610 nm for CHGO:0.075Eu^3+^ and CHGO:0.075Eu^3+^, 0.075M^+^ (M = Li, Na, K) phosphors.

As shown in Figure 5a, the XRD diagrams of CHGO:0.075Eu^3+^ and CHGO:0.075Eu^3+^, 0.075M^+^ (M = Li, Na, K) series samples are given. It can be seen from the diagrams that all samples are consistent with the standard cards, so the doping of charge compensator M^+^ (M = Li, Na, K) does not change the structure of CHGO:0.075Eu^3+^ phosphor. In addition, the intensity of the diffraction peak shows that the introduction of co-doped alkali metal ions M^+^ (M = Li, Na, K) can improve the crystallinity and particle size, and promote the phase formation and grain growth of matrix crystals [33]. Figure 5b shows the emission spectrum of CHGO:0.075Eu^3+^ under the action of different charge compensation ions M^+^ (M = Li, Na, K). Except for the obvious change in luminescence intensity, the shape and position of the emission peaks show no obvious change. Figure 5c shows the relationship between the emission intensity of the main fluorescence peak and the doping of different alkaline earth metal ions. As shown in the figure, after adding charge compensator M^+^ (M = Li, Na, K), the emission intensity of the sample has obviously increased. The increase in photoluminescence intensity is [K^+^] > [Li^+^] > [Na^+^], and the fluorescence intensity of CHGO:0.075Eu^3+^, 0.075 K^+^ is more than twice that of CHGO:0.075Eu^3+^ phosphors. Therefore, the doping of charge compensator M^+^ (M = Li, Na, K) can supplement the charge imbalance caused by vacancies, reduce lattice defects, effectively improve the activity of activation centers and enhance the luminescence efficiency [34].

**Figure 6:**
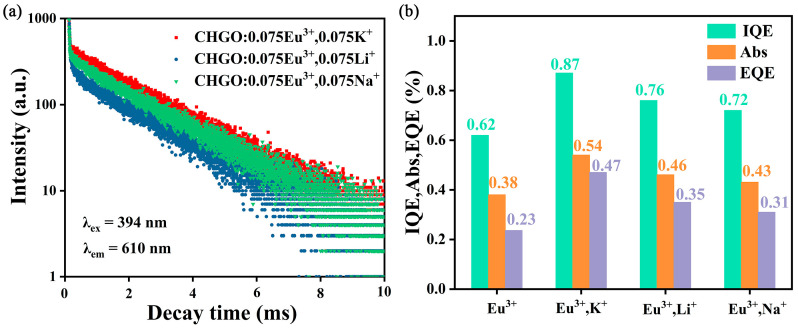
(**a**) The decay curves of CHGO:0.075Eu^3+^ and CHGO:0.075Eu^3+^, 0.075M^+^ (M = Li, Na, K) phosphors. (**b**) The IQE, Abs and EQE of CHGO:0.075Eu^3+^, CHGO:0.075Eu^3+^, 0.075Li^+^, CHGO:0.075Eu^3+^, 0.075K^+^ and CHGO:0.075Eu^3+^, 0.075Na^+^ phosphors.

Figure 6a shows the fluorescence decay curves of monitored samples CHGO:0.075Eu^3+^, 0.075Li^+^, CHGO:0.075Eu^3+^, 0.075K^+^ and CHGO:0.075Eu^3+^, 0.075Na^+^ at a wavelength of 610 nm under 394 nm light excitation. According to Equation (4), the lifetimes of the corresponding samples can be calculated as 1.35, 1.56 and 1.48 ms. Compared with the lifetime of CHGO:0.075Eu^3+^ sample, the lifetime of CHGO:0.075Eu^3+^ doped with charge compensator M^+^ (M = Li, Na, K) shows an increasing trend, which indicates that the probability of non-radiative energy transfer will be reduced and the fluorescence lifetime of Eu^3+^ will be increased when the charge compensator M^+^ (M = Li, Na, K) is doped with CHGO:0.075Eu^3+^ samples. In addition, quantum efficiency is an important index to evaluate the potential application of phosphors. As shown in Figure S2, the internal quantum efficiency (IQE), absorption efficiency (Abs) and external quantum efficiency (EQE) of the sample excited at 394 nm are obtained by using the integrating sphere technique. The calculation formulas are as follows [35,36]:

(6) $$IQE=\frac{\int L_{s}}{\int E_{R}-\int E_{s}}$$

(7) $$Abs=\frac{\int E_{R}-\int E_{s}}{\int E_{R}}$$

(8) EQE=Abs×IQE

Among them, L_s_ is the emission spectral intensity of the sample, and E_s_ and E_R_ are the excitation spectral intensities of the sample and BaSO_4_ reference sample, respectively. As shown in Figure 6b, IQE, Abs and EQE of CHGO:0.075Eu^3+^, CHGO:0.075Eu^3+^, 0.075Li^+^, CHGO:0.075Eu^3+^, 0.075K^+^ and CHGO:0.075Eu^3+^, 0.075Na^+^ samples are calculated. Detailed results are shown in Table 2. Among them, CHGO:0.075Eu^3+^, 0.075K^+^ sample shows the best performance, and its IQE is 87%, which is equivalent to that of commercial Y_2_O_3_:Eu^3+^ (63.40%), and far higher than that of Y_2_O_2_S:Eu^3+^ (35%) and other reported garnet red phosphors, such as Li_2_ZnTi_3_O_8_:Eu^3+^ (48.91%) and Ca_2_GaNbO_6_:Eu^3+^ (69.24%) [37,38].

### 2.4. Thermal Stability Analysis

**Figure 7:**
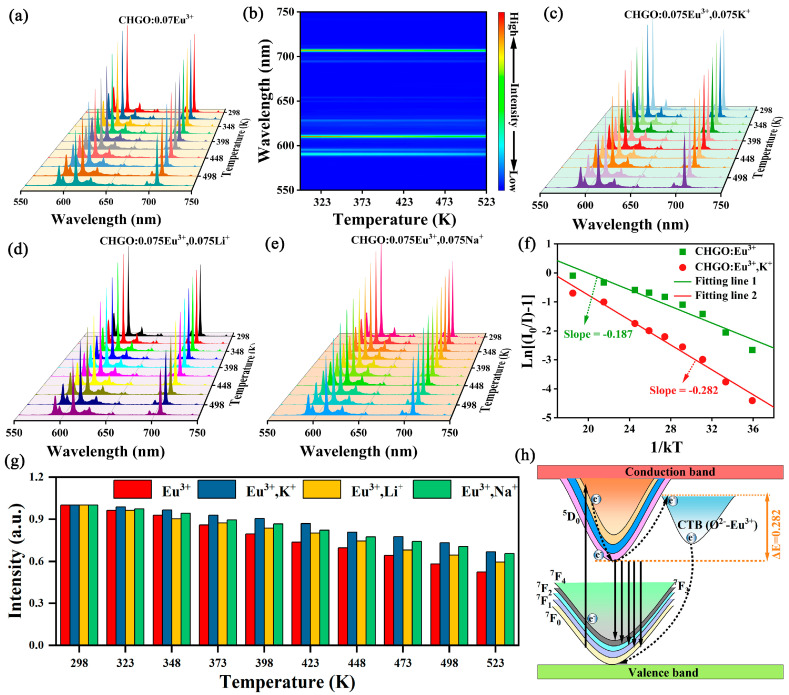
(**a**) Temperature-dependent emission spectra and (**b**) corresponding contour map of CHGO:0.075Eu^3+^. Temperature-dependent emission spectra of (**c**) CHGO:0.075Eu^3+^, 0.075K^+^, (**d**) CHGO:0.075Eu^3+^, 0.075Li^+^, (**e**) CHGO:0.075Eu^3+^, 0.075Na^+^. (**f**) The plot of ln[(I_0_/I_(T)_ − 1] VS. 1/KT for CHGO:0.075Eu^3+^ and CHGO:0.075Eu^3+^, 0.075K^+^ phosphors. (**g**) The integrated intensities of Eu^3+^ at 610 nm for CHGO:0.075Eu^3+^ and CHGO:0.075Eu^3+^, 0.075M^+^ (M = Li, Na, K) phosphors. (**h**) The schematic configuration coordinate diagram of Eu^3+^ for the explanation of temperature quenching.

In modern lighting technology, LEDs are favored because of their high efficiency, energy savings, and long life. However, LEDs will inevitably generate a large amount of heat in the working process, so that the device temperature may be as high as 420 K, which puts forward a high requirement for the thermal stability of fluorescent materials for pc-WLEDs [39]. In order to meet this challenge, the thermal stability of CHGO:0.075Eu^3+^ was studied in detail. Figure 7a–e and Figure S3 show the changes in emission spectra and corresponding contour maps of CHGO:0.075Eu^3+^, CHGO:0.075Eu^3+^, 0.075Li^+^, CHGO:0.075Eu^3+^, 0.075K^+^ and CHGO:0.07Eu^3+^, 0.075Na^+^ in the temperature range from 298 K to 523 K, respectively. It can be seen that with the increase in temperature, the luminescence intensity of all samples decreases, but the position and shape of the emission peaks of the samples are almost unchanged. Figure 7g is the relationship between normalized emission intensity and temperature at 610 nm of CHGO:0.075Eu^3+^, CHGO:0.075Eu^3+^, 0.075Li^+^, CHGO:0.075Eu^3+^, 0.075K^+^ and CHGO:0.07Eu^3+^, 0.075Na^+^ samples. It can be seen from the figure that compared with the CHGO:0.075Eu^3+^ sample, the doping of charge compensator M^+^ (M = Li, Na, K) effectively improves the thermal stability of the sample [40]. This is because the introduction of charge compensator M^+^ (M = Li, Na, K) effectively reduces the lattice distortion caused by charge imbalance when Eu^3+^ directly replaces Ca^2+^ in the CHGO:Eu^3+^ system, thus significantly improving the thermal stability of the samples. Among them, CHGO:0.075Eu^3+^, 0.075K^+^ showed the best results. Compared with its luminescence intensity at room temperature, the luminescence intensity at 423 K could still keep about 88.7%, which indicated that CHGO:0.075Eu^3+^, 0.075K^+^ had good thermal stability. In addition, the relationship between luminous intensity and temperature can be described by the following formula [41]:

(9) $$I_{\left(T\right)}=\frac{I_{0}}{1+cexp(-(E_{a}/kT))}$$

where I_0_ is the initial emission intensity, I is the emission intensity at temperature T, A is a constant, k is the Boltzmann constant, and ΔE is the activation energy of the thermal quenching process. As shown in Figure 7f, when ln (I_0_/I) is plotted with 1/kT, the slopes of CHGO:0.075Eu^3+^ and CHGO:0.075Eu^3+^, 0.075K^+^ samples after linear fitting are 0.187 and 0.282, respectively, that is, the activation energies are ΔE = 0. 187 eV and ΔE = 0. 282 eV, respectively. Higher ΔE indicates that the luminescent thermal stability of the sample is good. In addition, the thermal quenching mechanism of CHGO:0.075Eu^3+^ phosphor can be explained by the configuration coordinate diagram. As shown in Figure 7h, with the increase in temperature, after the electrons on ^5^D_J_ are excited, the potential barrier overcoming ΔE enters CTB through the intersection of ^5^D_J_ and CTB, and then the electrons relax to a lower energy level without radiation through the intersection of CTB and ^7^F, resulting in temperature quenching.

### 2.5. Applications in LEDs

**Figure 8:**
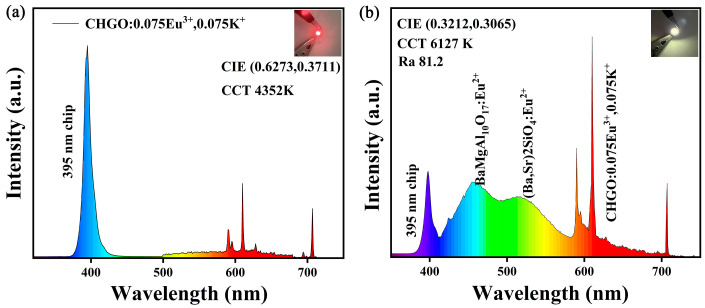
Electroluminescence spectra of the prepared (**a**) red LED and (**b**) white LED devices at 20 mA.

Based on the good thermal stability and color stability of the CHGO:0.075Eu^3+^, 0.075K^+^ sample, red and white LED devices were packaged to evaluate their potential applications. The electroluminescence spectrum, CIE color coordinates and CCT parameters of the device are shown in Figure 8a,b. Under a 20 mV current drive, both devices show a good luminescence effect. The CCT of the fabricated WLED device is 6127 K, Ra is 81.2, and its performance is slightly better than that of the common commercial products of YAG:Ce^3+^ blue chip combination in the market (CCT is about 7746 K, Ra value is 80). However, compared to high-performance LED devices on the market, there is still potential for further improvement in color purity. In addition, the CIE coordinates of the WLED device (0.3212, 0.3065) are located in the white light area, which is very close to the ideal white light equal energy point (0.333, 0.333) [42,43]. These excellent properties indicate that the CHGO:0.075Eu^3+^, 0.075K^+^ sample has broad application prospects in the field of solid-state lighting.

## 3. Materials and Methods

### 3.1. Preparation of Materials

The CHGO:xEu^3+^ (x = 0, 0.025, 0.05, 0.075, 0.1, 0.125, 0.15) and CHGO:0.075Eu^3+^, 0.075M^+^ (M = Li, Na, K) series fluorescent materials were prepared by a high-temperature solid-state method. The CaCO_3_ (98%, Aladdin, Shanghai, China), HfO_2_ (98%, Aladdin, Shanghai, China), Ga_2_O_3_ (99.9%, Aladdin, Shanghai, China), Eu_2_O_3_ (99.9%, Aladdin, Shanghai, China), Li_2_CO_3_ (98%, Aladdin, Shanghai, China), Na_2_CO_3_ (98%, Aladdin, Shanghai, China) and K_2_CO_3_ (98%, Aladdin, Shanghai, China) were used as raw materials, weighed in turn according to the corresponding stoichiometric ratio, and the above chemicals were ground in agate mortar for 1 h until they were mixed evenly. The mixture was then put into a corundum crucible and calcined at 1400 °C for 6 h. Finally, the sintered samples are naturally cooled to room temperature, and the next optical characterization is carried out.

### 3.2. Characterization of Materials

Its composition and crystal structure were measured using a Bruker D8 Focus X-ray powder diffractometer (XRD, Bruker, Billerica, MA, USA, λ = 0.15405 nm). The morphology and energy spectrum of the samples were analyzed by a field emission electron scanning microscope (SEM, S4800, Hitachi, Tokyo, Japan) equipped with an energy-dispersive spectroscopy (EDS) analyzer. The fluorescence excitation spectra, emission spectra and temperature-dependent spectra of the samples were measured by an Edinburgh Fluorescence Spectrometer (FLS1000) (Edinburgh, Livingston, UK), and the temperature-dependent spectra were measured by a temperature device (TAP-02) (Tian Jin Orient—KOJI Instrument, Tianjin, China). The fluorescence lifetime source was a high-energy pulsed xenon lamp. The electroluminescence properties of WLEDs were characterized by a photoelectric measurement system (HASS 2000) (Everfine, Hangzhou, China) with an integrating sphere spectroradiometer system.

## 4. Conclusions

In summary, a series of CHGO:xEu^3+^ (x = 0, 0.025, 0.05, 0.075, 0.1, 0.125, 0.15) and CHGO:0.075Eu^3+^, 0.075M^+^ (M = Li, Na, K) samples were prepared via the high-temperature solid-state method. The XRD test results indicate that the doping of Eu^3+^ and charge compensator M^+^ (M = Li, Na, K) has not altered the crystal structure of the CHGO material. Under the excitation of 394 nm, the emission spectrum of CHGO:0.075Eu^3+^ phosphor shows five emission peaks, which belong to the ^5^D_0_→^7^F_J_ (J = 1, 2, 3, 4) energy level transitions of Eu^3+^. Under the monitoring of 610 nm, the strongest excitation peak of the phosphor is located at 394 nm, which belongs to the ^7^F_0_→^5^L_6_ energy level transition. When the doping concentration of Eu^3+^ reaches x = 0.075, the sample exhibits the best luminescence performance, and a higher doping concentration will lead to concentration quenching. In addition, with the increase in Eu^3+^ doping concentration, the fluorescence lifetime of the phosphor decreases due to the increase in non-radiative energy transfer. The incorporation of the charge compensator M^+^ (M = Li, Na, K) is beneficial to eliminate the interstitial oxygen defect caused by the valence compensation, thereby improving the luminescence intensity, fluorescence lifetime and thermal stability of the phosphor. Finally, the thermal stability of the phosphor CHGO:0.075Eu^3+^, 0.075K^+^ was investigated, exhibiting a luminous intensity at 423 K of approximately 88.7% relative to its room-temperature luminous intensity. Consequently, the CHGO:0.075Eu^3+^, 0.075K^+^ phosphor represents a promising candidate material for display devices and white light LED applications.

## Figures and Tables

**Table 1:** Refinement results of CHGO, CHGO:0.025Eu^3+^, CHGO:0.075Eu^3+^, and CHGO:0.125Eu^3+^ samples.

Sample	CHGO	CHGO:0.025Eu^3+^	CHGO:0.075Eu^3+^	CHGO:0.125Eu^3+^
Space group	Ia-3d	Ia-3d	Ia-3d	Ia-3d
Symmetry	cubic	cubic	cubic	cubic
a/b/c, Å	12.5661	12.5423	12.4835	12.4522
V, Å^3^	1984.27	1972.88	1945.17	1930.71
Z	8	8	8	8
α = β = γ °	90	90	90	90
R_wp_	7.54	8.55	8.02	8.69
R_p_	5.71	5.90	5.44	6.45
χ^2^	2.13	3.58	2.53	3.21

**Table 2:** Lifetimes, IQE, Abs and EQE of CHGO:0.075Eu^3+^, CHGO:0.075Eu^3+^, 0.075Li^+^, CHGO:0.075Eu^3+^, 0.075K^+^ and CHGO:0.075Eu^3+^, 0.075Na^+^ phosphors.

Sampe	Lifetime (ms)	Abs (%)	IQE (%)	EQE (%)
CHGO:0.075Eu^3+^	1.23	38.3	62.5	23.6
CHGO:0.075Eu^3+^, 0.075Li^+^	1.35	46.2	76.1	35.1
CHGO:0.075Eu^3+^, 0.075K^+^	1.56	54.7	87.0	47.0
CHGO:0.075Eu^3+^, 0.075Na^+^	1.48	43.6	72.3	31.1

## Data Availability

The research data are available from the authors on request.

## Supplementary Materials

The following supporting information can be downloaded at: https://www.mdpi.com/article/10.3390/molecules31132397/s1, Figure S1. XRD Rietveld refinement of (a) CHGO:0.025Eu^3+^, (b) CHGO:0.05Eu^3+^, (c) CHGO:0.1Eu^3+^, (d) CHGO:0.125Eu^3+^ and (e) CHGO:0.15Eu^3+^; Figure S2. Excitation line of BaSO_4_ and the emission spectrum of (a) CHGO:0.075Eu^3+^, (b) CHGO:0.05Eu^3+^, 0.075K^+^, (c) CHGO:0.05Eu^3+^, 0.075Na^+^, and (e) CHGO:0.05Eu^3+^, 0.075Li^+^ phosphors; Figure S3. Temperature-dependent emission spectra of (a) CHGO:0.05Eu^3+^, 0.075K^+^, (b) CHGO:0.05Eu^3+^, 0.075Li^+^ and (c) CHGO:0.075Eu^3+^,Na^+^.
