# Supplementary material for: Charge Compensation-Directed Enhanced Photoluminescence in M+ (M = Li, Na, K) Co-Doped Novel Red Phosphor Ca2.5Hf2.5Ga3O12:Eu3+ for Lighting Applications
Source: Molecules. 2026 Jul 7;31(13):2397. doi: 10.3390/molecules31132397 (PMC13363428; doi:10.3390/molecules31132397)
Supplement: Supplementary file 1 [file molecules-31-02397-s001.zip › molecules-4345432-supplementary.pdf]

Supporting information

Charge Compensation-directed Enhanced Photoluminescence in M^+ (M = Li, Na, K) Co-doped Novel Red Phosphor $Ca_{2.5}Hf_{2.5}Ga_3O_{12}$:$Eu^{3+}$ for Lighting Applications

Hua Li *, Zijun Huang, Yifei Hou, Qiyue Liu, Di Li, Wenyue Zhang, Yi Su, Zhide Wang and Zaifa Yang *

College of Physics and Electronic Engineering, Qilu Normal University, Jinan, 250200, PR China

*Correspondence: hualiqlnu@126.com (H. Li); yangzaifa@qlnu.edu.cn (Z.F. Yang)

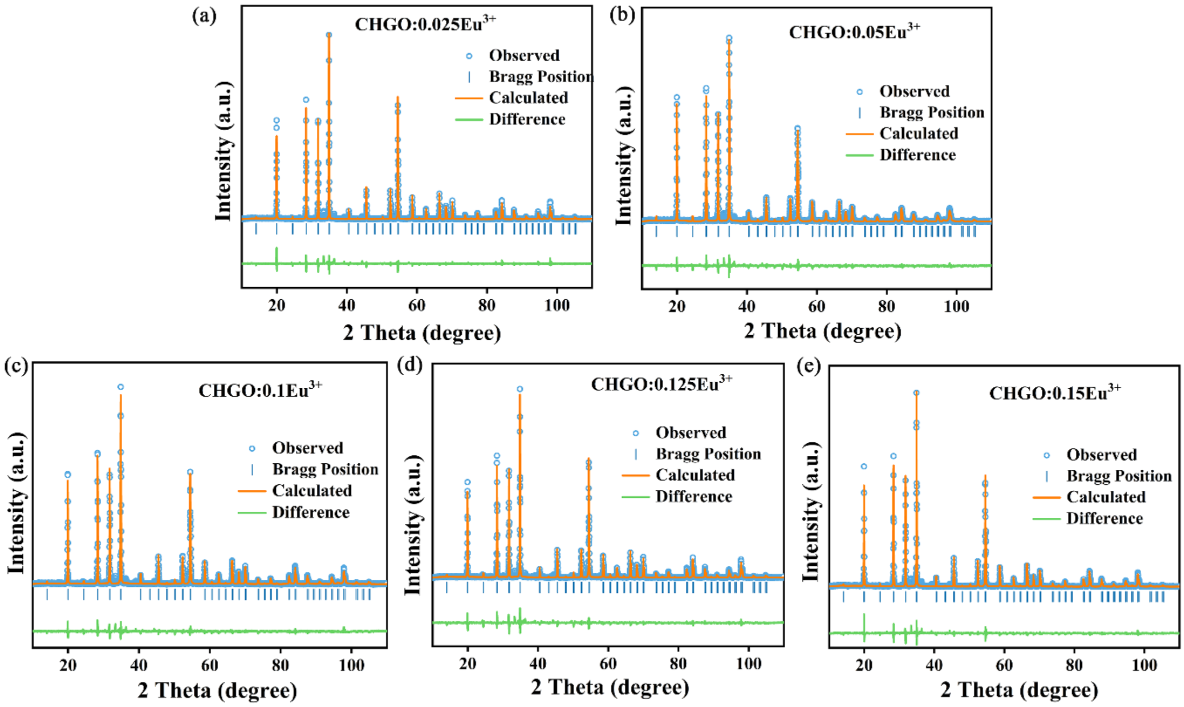

Figure S1 XRD Rietveld refinement of (a) CHGO:0.025Eu^{3+}, (b) CHGO:0.05Eu^{3+}, (c) CHGO:0.1Eu^{3+}, (d) CHGO:0.125Eu^{3+} and (e) CHGO:0.15Eu^{3+}.

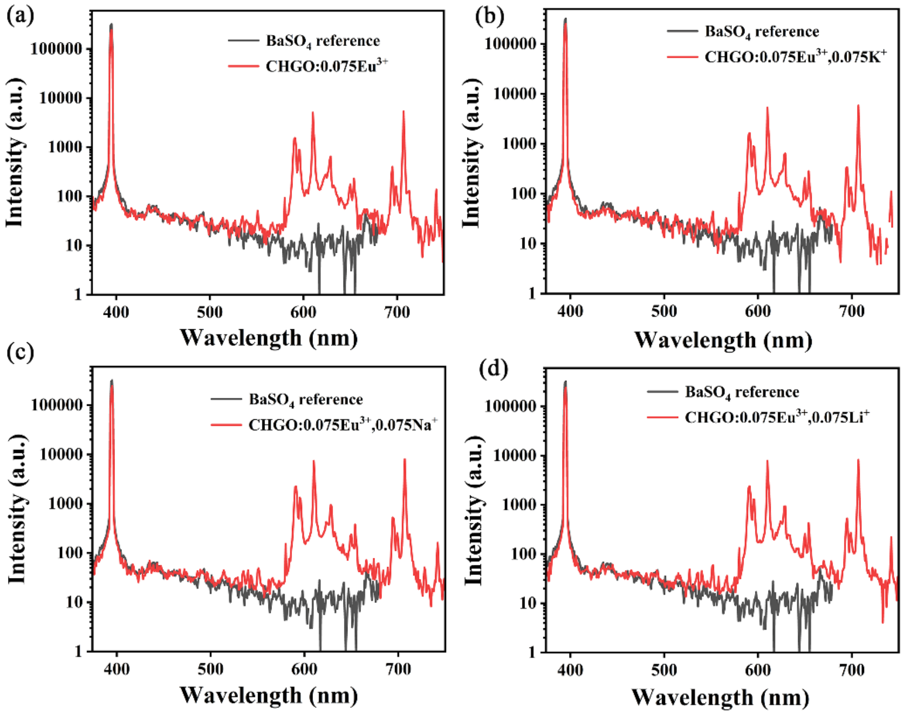

Figure S2 Excitation line of $BaSO_4$ and the emission spectrum of (a) CHGO:0.075Eu^{3+}, (b) CHGO:0.05Eu^{3+},0.075K^{+}, (c) CHGO:0.05Eu^{3+},0.075Na^{+}, and (e) CHGO:0.05Eu^{3+},0.075Li^{+} phosphors.

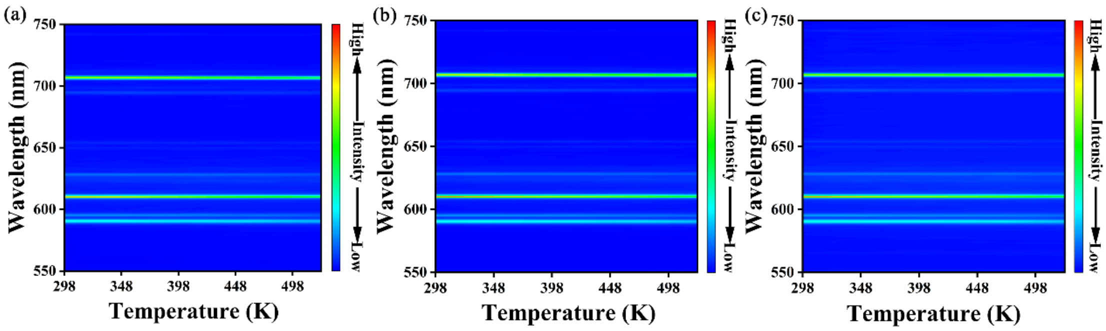

Figure S3 Temperature-dependent emission spectra of (a) CHGO:0.05Eu^{3+},0.075K^{+}, (b) CHGO:0.05Eu^{3+},0.075Li^{+} and (c) CHGO:0.075Eu^{3+},Na^{+}.
